# In situ cryo-electron tomography reveals gradient organization of ribosome biogenesis in intact nucleoli

**DOI:** 10.1038/s41467-021-25413-w

**Published:** 2021-09-10

**Authors:** Philipp S. Erdmann, Zhen Hou, Sven Klumpe, Sagar Khavnekar, Florian Beck, Florian Wilfling, Jürgen M. Plitzko, Wolfgang Baumeister

**Affiliations:** 1 Max Planck Institute of Biochemistry, Martinsried, Germany; 2 Fondazione Human Technopole, Milano, Italy; 3 Max Planck Institute of Biophysics, Frankfurt, Germany

**Keywords:** Nucleus, Cryoelectron tomography

## Abstract

Ribosomes comprise a large (LSU) and a small subunit (SSU) which are synthesized independently in the nucleolus before being exported into the cytoplasm, where they assemble into functional ribosomes. Individual maturation steps have been analyzed in detail using biochemical methods, light microscopy and conventional electron microscopy (EM). In recent years, single particle analysis (SPA) has yielded molecular resolution structures of several pre-ribosomal intermediates. It falls short, however, of revealing the spatiotemporal sequence of ribosome biogenesis in the cellular context. Here, we present our study on native nucleoli in *Chlamydomonas reinhardtii*, in which we follow the formation of LSU and SSU precursors by in situ cryo-electron tomography (cryo-ET) and subtomogram averaging (STA). By combining both positional and molecular data, we reveal gradients of ribosome maturation within the granular component (GC), offering a new perspective on how the liquid-liquid-phase separation of the nucleolus supports ribosome biogenesis.

## Abstract (web summary)

The large and small subunits of the ribosome are synthesized independently within the nucleolus — a membrane-less compartment within the nucleus — before being exported into the cytoplasm. Here, the authors use in situ cryo-ET to observe ribosome maturation and reveal the native organization of the nucleolus.

## Introduction

The assembly of eukaryotic ribosomes is a complex process involving up to 200 proteins and RNAs^1^, which are transcribed from a single copy of ribosomal DNA (rDNA). Purified and spread rDNA (Miller spreads) have been used to investigate the nuclear stages of pre-ribosome formation in vitro. This has enabled the structural identification of both the active polymerase (Pol I) as well as “terminal knobs”, which have been suggested to correspond to SSU and LSU precursors^2–4^. In cells, these early steps of ribosome biogenesis take place in a dedicated nuclear compartment, the nucleolus, which has been studied extensively using fluorescence and conventional plastic-embedded electron microscopy (EM)^5^. Since nucleoli are not held together by a membrane, their isolation, although feasible^6^, poses a problem for studying their natural organization by cryo-electron microscopy (cryo-EM), which promises much improved structural preservation and resolution compared to conventional EM.

Several key aspects of nuclear ribosome biogenesis have recently been elucidated by high resolution SPA of various pre-ribosomal particles in different species^7–16^. While these atomic models have provided valuable insights, they cannot capture the spatiotemporal organization of ribosome maturation. Moreover, the need to stabilize and enrich intermediates via genetic or biochemical manipulation, could artificially favor certain states over others.

To overcome these limitations and to reconcile the wealth of morphological and molecular information available, we turned to in situ cryo-ET^17^ and visual proteomics^18^ to investigate the native organization of the nucleolus and its role in ribosome biogenesis.

## Results and discussion

Cryo-focused ion beam (FIB) milling allows preparation of electron-transparent samples preserved in their native state by cryo-fixation without the need for chemical agents and without the artifacts known from cryo-sectioning^19,20^. Traditionally, ribosome biogenesis has been investigated in the yeast *Saccharomyces cerevisiae* (*S. cerevisiae*) because of its robustness and the availability of genetic tools^21^. However, the localization of *S. cerevisiae* nucleoli can be technically challenging in a pure cryogenic workflow due to their unpredictable cellular location^22,23^. We therefore chose the green alga *Chlamydomonas reinhardtii* (*C. reinhardtii*) as it has an almost deterministic cellular architecture, eliminating the need for correlative targeting^24^. Although *Chlamydomonas* might have a simpler ribosome assembly pathway than other eukaryotes^25^, many biogenesis factors are conserved. Moreover, *C. reinhardtii* shows all the hallmark features common to nucleoli: At its center, the fibrillar and dense fibrillar components (FC and DFC), site of rDNA transcription, can be identified. Both are surrounded by the granular component (GC), where pre-ribosome assembly is thought to take place.

**Fig. 1:**
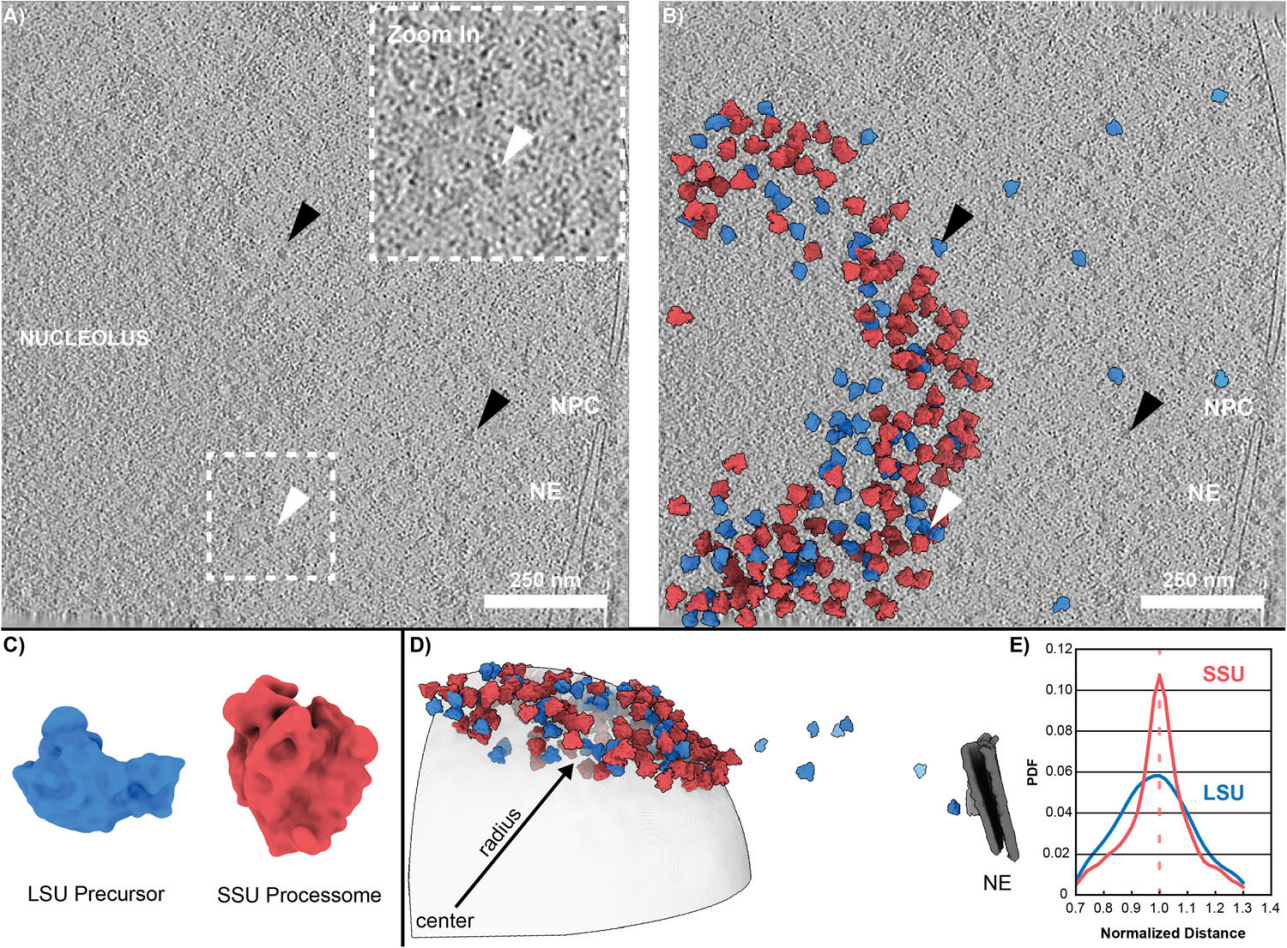
Structural and molecular organization of the C. reinhardtii nucleolus. **A** The C. reinhardtii nucleolus is clearly visible as a dense structure decorated with large particles (white arrow & zoom in). Some of these particles (black arrows) are also found floating in the nucleoplasm. **B**, **C** Using reference-free template matching, pre-60S particles (blue) and SSU processomes (red) are detected (*n* = 85 tomograms). **D** Their 3D organization is consistent with a sphere decorated by pre-ribosomes (*n* = 26 tomograms for which spheres could be fit). **E** The small subunit precursors show a characteristically different probability density function (PDF) than the pre-60S, with a sharper peak around the nucleolar surface (*n* = 26 tomograms; Fisher-Pitman permutation test^60^ with significantly smaller variance for the SSU: *p* = 1.0E–06). LSU Large Subunit, SSU Small Subunit, NE Nuclear envelope, NPC Nuclear Pore Complex. Source data are provided as a Source Data file.

To resolve their nucleolar organization, we subjected *Chlamydomonas* to our cryo-FIB workflow and obtained a large number of suitable tomograms slicing individual nucleoli at various angles and heights (Supplementary Fig. 1). Overall, the data shows the expected tripartite organization (Supplementary Fig. 1J, K). Additionally, owing to their native preservation, individual particles can now be distinguished. While most of the larger complexes appear to be confined to the nucleolus (Fig. 1A, white arrow), a fact also reported by conventional EM^26^, a small number of large, electron-dense particles is found in the nucleoplasm (Fig. 1A, black arrows). Beyond this qualitative assessment, template matching was used to search for and identify specific targets within the 3D data^27,28^. Using an unstructured reference (Supplementary Fig. 2), the pre-60S and SSU processome were found as the two dominant types of nucleolar complexes (Fig. 1B, C, Supplementary Movie 1). While the small subunit precursor seems mostly restricted to the nucleolus, some LSU-like complexes are also detected far out in the nucleoplasm (Fig. 1B), which is consistent with their expected remodeling prior to export to the cytosol^15,16,29^.

In tomograms that covered a sufficiently large area, we observed a distinct orbital pattern of pre-ribosomal particles around the nucleolus (Supplementary Fig. 2). We therefore devised an algorithm that fits spheres to their combined 3D positions to determine the size and centers of the nucleoli (Fig. 1D and Supplementary Fig. 3). At 599 ± 29 nm (mean ± SEM; *n* = 26 tomograms), their mean radius agrees with previous studies^30^. The individual probability distribution functions (PDFs), however, reveal distinct localization patterns of pre-60S and SSU-processome complexes (Fig. 1E). While both show an increase in particle number with distance from the center, the SSU precursor has a significantly sharper peak around the calculated mean radius - hereafter referred to as the “surface” of the nucleolus. This gradient architecture is intriguing because the particles seem to delineate the very transition from the GC to the nucleoplasm. While numerous studies have attempted to assign functional roles to individual nucleolar components and to localize specific ribosomal precursors within them, it is still unclear whether the layered FC/GC organization is required for ribosome formation or rather a consequence thereof. Nevertheless, there is evidence that formation of the rRNA transcripts is key to the formation and maintenance of nucleolar organization^31^. Unlike other organelles, nucleoli are not surrounded by a membrane and arise from liquid-liquid phase separation (LLPS). Recent studies have shown that LLPS is responsible for the assembly of the dense fibrillar component in the human nucleolus^32^, and that the FC-GC core-shell architecture of *Xenopus laevis* oocytes, is based on the surface tension of the individual protein components^33^. Although heterotypic multicomponent interactions have been suggested to drive cellular liquid-liquid phase separation^34^, it remains unclear how such an organization is maintained on a per-particle basis.

Given the differential localization of LSU and SSU precursors, we decided to further explore the nucleolar architecture in *Chlamydomonas*. While numerous smaller particles can be observed toward the center of the nucleoli (Fig. 1A and Supplementary Fig. 1), template matching with unstructured references of different sizes did not yield any additional well-defined averages. This might be due to the dynamic nature of pre-ribosomes and certain experiment- and sample-related limitations, *e.g*. particle number, pixel size, and SNR. In combination, these factors might preclude robust classification and thereby identification of smaller particles such as the pre-40S.

**Fig. 2:**
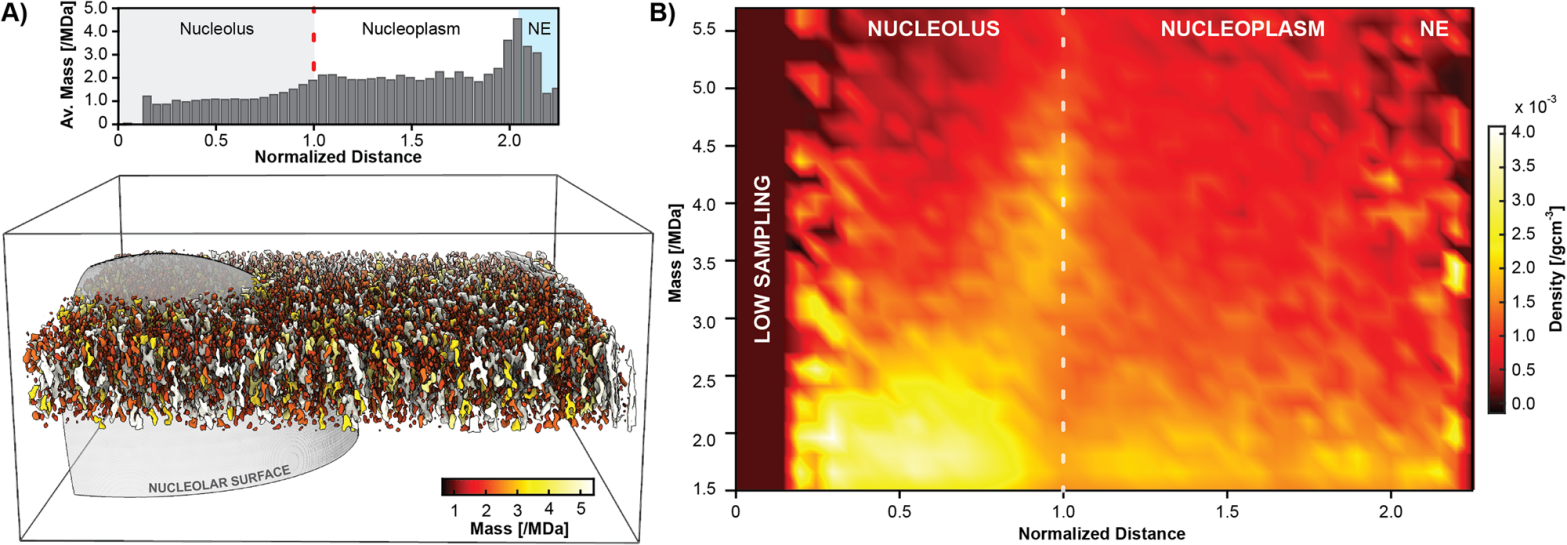
Image-based analysis of the nucleolar organization. **A** Individual tomograms can be denoised^50^ and filtered to allow segmentation of nuclear particles (bottom). Analysis of their apparent mass (top) shows an increase in apparent mass per particle toward the surface of the nucleolus starting at ~0.75 of the normalized distance. Before and after that, the average mass remains relatively constant until the nuclear envelope is reached. **B** Spectral analysis of particle mass vs. normalized distance over all tomograms with sphere fits shows two main features: 1. There are constantly high levels of particles up to a size of ~2.5 MDa, except around the surface of the nucleoli (dashed line). 2. At this distance, a lack of smaller masses is compensated by a burst of large complexes, consistent with the detection of pre-ribosomal particles such as the SSU processome and the pre-60S. NE Nuclear envelope. Source data are provided as a Source Data file.

To overcome these limitations of STA, we turned to an approach based solely on image analysis. Densities within each tomogram were segmented and their average size was determined after denoising and filtering (See Methods for details). With the nucleolar centers as reference points, we performed a distance analysis, finding that, on average, the apparent particle mass increases throughout the granular component, with an initial maximum at the surface of the nucleolus where most pre-60S and SSU processomes are detected (Fig. 2A, top).

To further corroborate this finding, we performed an analysis of the particle density as a function of mass and distance from the nucleolar center. This revealed two major properties: an almost constant background of smaller particles up to ~2.5 MDa, and a spike of larger complexes (3–5.5 MDa) toward the surface of the nucleolus (Fig. 2B; dashed line). This suggests that GC particles, which at this point lack an exact identity, are partitioned by size and thus potentially by developmental stage.

**Fig. 3:**
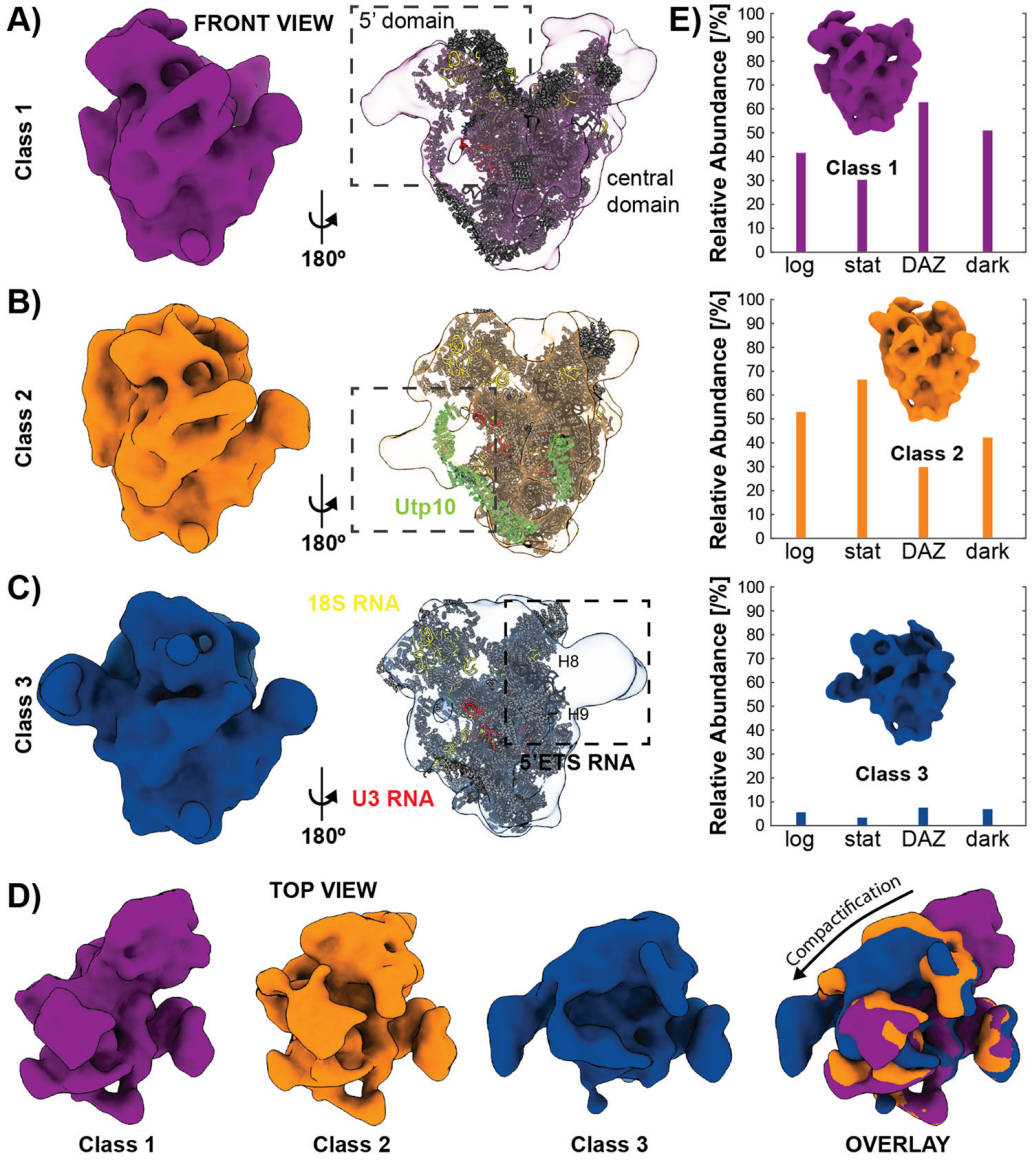
Structural heterogeneity of in situ SSU processome particles. **A**–**C** Three distinct SSU processome class averages (Class 1–3) with different similarity to published structures are detected. **D** There is a clear trend from Class 1 to Class 3 toward a more compact arrangement of the 5′ and central domains. This is particularly evident when all three STA structures are aligned and superimposed, and indicates a sequence of maturation (Supplementary Movie 2). **E** The relative abundance of each class is subject to external stimuli. While Class 2 dominates during logarithmic (log) and stationary growth (stat), Class 1 becomes the predominant form when treated with diazaborine (DAZ), a drg1 AAA-ATPase inhibitor, and when ribogenesis is stimulated (dark). PDB model for **A**–**C**: 5WYJ. See Supplementary Fig. 6 for a structural comparison with other maps. Source data are provided as a Source Data file.

We therefore hypothesized, that this might also be true for the later stages, where averages can be obtained and thus identities can be assigned. To test this idea, we performed additional rounds of template matching, this time using the previously obtained, well-structured averages as starting points. In order to avoid reference bias, a template-truncation strategy was implemented^35^, since otherwise false positive rates can be inflated (Supplementary Fig. 4A). Based on the new global averages, sub-classification (Supplementary Fig. 4B) was performed for both precursors. For the pre-60S, we found three major classes that resemble late nucleolar and nucleoplasmic intermediates from published SPA studies^12^, which validates our classification approach (Supplementary Fig. 8A–C). For the small subunit processome, there has been little information on conformational heterogeneity until recently when additional intermediates were described using SPA and refined isolation and preparation techniques^36,37^. Since our in situ approach does not require any biochemical treatments, it provides an unbiased view of all intermediates in their native context. After implementing a rigorous classification strategy and checking for cross-effects (Supplementary Fig. 5), we found three distinct SSU processome classes (Fig. 3A–C). Despite their moderate resolution (~25 Å), large domains and their movement between states can still be interpreted and compared to single particle results. Overall, our in situ structures are in good agreement with published SPA maps (Supplementary Fig. 6A–C)^8,10,38^, including iconic features such as helix 44 and the base region of UtpA, which is formed by the beta-propellers of Utp17 and Utp8^38^. However, unlike the single particle structures, some densities seem to be more continuous (Supplementary Movie 2) and there are features which are not present in the SPA maps. While these certainly could represent *Chlamydomonas*-specific subunits, they could also correspond to cofactors lost during purification for single particle cryo-EM.

Some of the observed variance could be due to both lower resolution and species-specific subunits. However, the small changes reported so far between organisms that are evolutionarily quite distant is more indicative of physiologically relevant structural features^8,38^. Of the three, SSU processome Class 2 shows the highest similarity to published maps overall (Supplementary. Fig. 6D), except for an additional density found around the location of UTP10 (Fig. 3B). Contrarily, Classes 1 and 3 appear significantly different and hence merit further investigation.

Compared to the available SPA-structures, Class 1 with its 5′ domain distinctly shifted away from the central domain (Fig. 3A) displays a more open conformation than published SSU processome intermediates. It is therefore reasonable to assume that Class 1 represents an early state. In contrast, Class 3, the least abundant class, is fairly similar to published structures with respect to both orientation and location of the 5′ domain, albeit slightly more compact. Additionally, there is a prominent density next to Utp14 at helix 8 and 9 within the 5′ETS RNA, the suggested binding site of MTR4 and by proxy the nuclear exosome (Fig. 3C). Comparing all three maps, there is an apparent compactification from Class 1–3, during which the 5′ moves closer to the central domain and therefore its mature state (Fig. 3D). Finally, comparing our structures to SSU intermediates recently solved by SPA (Supplementary Fig. 6E)^36,37^ shows that the order of Class 1–3 is consistent with progressing stages of maturation. Class 1 even seems to pre-date the earliest isolated structures so far and Class 3 corresponds to a late, exosome-bound intermediate (Supplementary Fig. 6F, G).

To further substantiate this proposed order, we examined the relative abundance of SSU intermediates in *Chlamydomonas* cells that were subjected to different stimuli (Fig. 3E). During log phase, SSU Class 1 and 2 are detected at 42% and 53%, respectively, while Class 3 makes up only 5%, potentially suggesting a short-lived intermediate. Under stationary growth conditions when nutrients are limited and consequently the rate of ribosome biogenesis is low, Class 2 becomes the predominant form by far (66%). Interestingly, this also matches experimental conditions of earlier SPA studies and hence might explain the strongest similarity of Class 2 with these maps (Supplementary Fig. 6D). An opposite shift in abundance is observed when cells are treated with diazaborine (DAZ), a drug known to interfere with late-stage pre-60S biogenesis. DAZ inhibits the AAA-ATPase drg1 and in turn prevents the release and recycling of Rlp24 after shuttling the LSU precursor to the cytoplasm^39,40^. Brief DAZ-treatment of *C. reinhardtii* cells shifts both intermediates of the large and the small subunit toward the earlier states. While diazaborine has been reported to selectively affect the pre-60S^39^, which we also capture in the classification of our LSU precursors (Supplementary Fig. 7F), it is conceivable that the failure to recover important late-stage nucleolar LSU factors could also lead to changes in the biogenesis of the SSU processome. Alternatively, a lack of functional cytosolic 60S subunits could initially stimulate ribosome biogenesis and hence result in an increase of early intermediates. As ribosome formation in *Chlamydomonas* is linked to its day/night cycle, we prepared synchronized cultures and harvested the cells 4 h after dark phase initiation, the expected peak of ribogenesis^41^. This physiological stimulus again shifts the prevalence in favor of Class 1, making it the major SSU precursor at 52%, followed by Class 2 at 42%, and Class 3 at 6%.

**Fig. 4:**
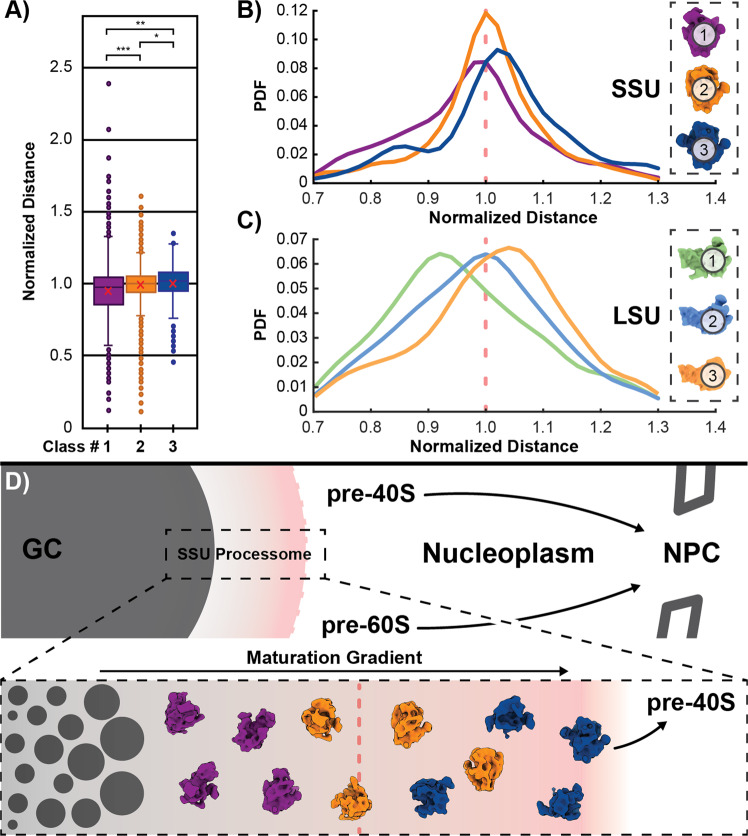
Sub-Nucleolar Partitioning of Pre-Ribosomes. **A** The SSU processome classes show significant differences in their spatial distribution depending on their level of maturation (*n* = 26 tomograms; two-sided Fisher-Pitman permutation test^60^ of mean: *p*_1,2_ = 1 E−6, *p*_1,3_ = 1.07 E−04, *p*_2,3_ = 4.1 E−02; *n* = 1809 particles; data are represented as boxplots where the red cross indicates the mean, the middle line is the median, the lower and upper hinges correspond to the first and third quartiles, top and bottom whiskers indicate maximum and minimum, respectively. Circles show outlier points. See Supplementary Fig. 7d for pre-60S results. **B**, **C** Plotting the probability density function (PDF) for the large and small subunit precursors reveals discrete behaviors. There is a sharp peak for SSU Class 2 & 3 at the nucleolar surface and Class 1 displays higher probabilities at smaller radii. Likewise, pre-60S Class 1 shows a peak at even lower distances, implying that particles can still be detected further inside the nucleolus and that template matching is not restricted to its periphery (*n* = 26 tomograms). **D** The data suggests a model, in which pre-ribosomal particles are separated in maturation gradients and thus provide molecular order to the nucleolus and ribosome biogenesis. SSU Small subunit, LSU large subunit, GC Granular Component, NPC Nuclear Pore Complex. Source data are provided as a Source Data file.

Having confirmed that SSU Classes 1–3 represent snapshots of progressive maturation, we checked for signs of spatial segregation, which would support our prior notion of a gradient organization within the GC. Focusing on the later stages, where STA averages can be obtained, we compared the normalized nucleolar center-to-particle distance of each individual complex, revealing a statistically significant increase in the mean from SSU Class 1 to 2, and 3 (Fig. 4A). To investigate this distribution in more detail, we plotted the probability density function for each class, exposing gradients with individual peaks for each intermediate (Fig. 4B, C). Combined with the previous results, our analysis therefore supports the idea of progressive maturation of pre-ribosomes within the granular component. In addition, the two peaks for SSU Class 3 PDF (Fig. 4B) may indicate alternate routes of exosome-binding and hence pre-rRNA processing, as has been proposed for several eukaryotic species^42^.

How do these findings fit into the larger picture of nucleolar organization, ribosome biogenesis, and LLPS? Our data suggest a model, in which starting from the center of the nucleolus, pre-ribosomal particles are processed successively, advancing both in size and maturation as they move radially outward until structurally well-defined intermediates appear. Furthermore, we show that the small subunit precursor in particular acts as a marker that defines the transition from the nucleolus to the nucleoplasm and hence from a liquid-liquid phase separated to a “normal” solution state. At this boundary, the SSU processome, which merely acts as a construction scaffold for the pre-40S particle, falls apart and is therefore not found much further beyond this point (Fig. 4D). Our data provides the first molecular-level evidence for theoretical work, which predict that the liquid-liquid phase separation organization of the nucleolus is a result of concentration gradients and the multivalent interactions of the pre-ribosomal complexes. As particles mature, it is suggested that sites for processing and thus for interaction with cofactors localized to the nucleolus are progressively lost. Accordingly, the multivalence of interactions between pre-ribosomes and the LLPS environment decrease. A maturation gradient is therefore expected before particles are eventually driven out of the liquid phase-separated domain^34^. This idea is clearly supported by our analysis of particle size and molecular identity of the SSU processome and the pre-60S, providing evidence for the partitioning of key processing steps within the nucleolus. Moreover, the distinct orbital pattern, which results from the final stages of nucleolar pre-ribosome assembly, shows that LLPS-like organelles, despite lacking membranes, can still coordinate and thus control biochemical reactions at interfaces similar to their “classical” counterparts. Controlling the dissociation of the particles from the LLPS could furthermore represent an elegant way to synchronize ribosome biogenesis. If the release “signal” is an intrinsic characteristic of the intermediates, this could serve as an integrated form of quality control. Clearly, larger datasets are required to decipher the finer details of this sub-nucleolar organization. This includes analysis in other organisms, identification of the smaller GC particles that have not yet been identified, and localization of other intermediates suggested by SPA studies like the 5′ETS^14^. Nevertheless, our current results already show that the nucleolus creates an environment which helps to separate crucial events of ribosome biogenesis both in space and time.

## Methods

### Cell culture

Cells used in this study were *Chlamydomonas reinhardtii* strain mat3-4 (CC3994) acquired from the Chlamydomonas Resource Center, University Minnesota, MN, which produces a smaller growth phenotype^43^. Unless otherwise stated, cells were agitated at 100 rpm.

#### Log phase

From a plate culture, a liquid culture was inoculated in 40 mL Tris-acetate phosphate (TAP) media (0.02 M Tris base, TAP salts (7.00 mM NH_4_Cl, 0.83 mM, MgSO_4_, 0.45 mM CaCl_2_), Phosphate buffer (1.65 mM K_2_HPO_4_, 1.05 mM KH_2_PO_4_), Hunter trace elements (0.134 mM Na_2_EDTA, 0.136 mM ZnSO_4_, 0.184 mM H_3_BO_4_, 40 µM MnCl_2_, 32.9 µM FeSO_4_, 12.3 µM CoCl_2_, 10.0 µM CuSO_4_, 4.44 µM (NH_4_)_6_MoO_3_, 17.5 mM acetic acid)). Cells were constantly illuminated (~90 µmol photons/m^2^s), aerated with normal atmosphere, and harvested 48–72 h after inoculation.

#### Stationary phase

A log phase culture was allowed to continue growing for seven days without dilution.

#### DAZ treatment

Log phase cells were treated with diazaborine (DAZ, 370 µM, Merck) for 2 h before plunge freezing.

#### Synchronization experiments

From a log-phase culture (24 h), a sample was taken and transferred to 40 mL of fresh TAP media. Cells were subsequently cultured in an isolated grow box with electrically controlled illumination using a 12 h/12 h day/night cycle. Every 2-3 days, the cell density was adjusted during the day period to ~1000 cells/µL by manually diluting the sample with fresh TAP and removing excess media and cells. This was maintained for three such cycles. Finally, cells were harvested after 4 h of initiating the dark cycle.

### Plunge freezing

A total of 4 µL of cell suspension (1500–2000 cells/µL) were applied to holey carbon R2/1 copper, or holey SiO_2_ R1/4 TEM grids (Quantifoil), and cells were plunge frozen on a Vitrobot Mark IV (FEI, settings: blotforce = 10, blottime = 10 s, temperature = 30 °C, humidity = 90%) using back-side blotting only and using an ethane/propane bath. Samples were stored under liquid nitrogen until use.

### FIB milling

Lamellas were prepared on a dual beam focused ion beam microscope (Quanta 3D FEG, FEI) equipped with a Quorum PP3000T cryo-system (Quorum Technologies, Laughton, United Kingdom) and a homemade 360° cryo-stage cooled by an open nitrogen circuit. Lamellas were cut from randomly selected, mostly individual *C. reinhardtii* cells analogous to published protocols^44^.

### Cryo-TEM

Cryo-tomograms were acquired on a transmission electron microscope (Titan Krios, FEG 300 kV, FEI) with a post-column energy-filter (968 Quantum K2, Gatan) with a defocus range of −5 µm to −3.5 µm and an EFTEM magnification of 42000x (calibrated pixel size 3.42 Å). Images were recorded with a direct detection camera (K2 Summit, Gatan) in dose-fractionation mode and a total dose of ~120 e^–^ / Å^2^ per tomogram using SerialEM^45^. The acquisition was controlled by an in-house script running a dose-symmetric tilt scheme^46^ with an angle increment of 2° between the range of 70° and − 50° starting at 10° to compensate for the lamella pre-tilt (~12°). In addition to the dose symmetric tilt schemes, 21 out of the total 85 tomograms were recorded using a bi-directional scheme with an angular increment of 2° starting at –20° and going to +70°. This was followed by a second branch from –22° to –50° to complete the series. Frames were aligned using motioncorr2^47^, the contrast transfer function (CTF) estimated using CTFFIND4^48^, stacks were assembled using an in house Matlab (MathWorks) script, and tilt-series alignment as well as tomogram reconstruction was performed in IMOD^49^ using custom Matlab (MathWorks) wrapper scripts for batch pre-processing. To avoid bias, tomogram names were hashed to conceal the experimental state (log, stationary, DAZ-treated, synchronized-dark; see above).

### Visualization of tomograms

To enhance the contrast of TEM insets, tomograms were denoised using TOPAZ^50^.

### Bias-mitigated template matching, classification & subtomogram averaging

To construct a featureless sphere, 300 nucleolar particles were manually picked and averaged in PyTOM^51^ with randomly assigned angles. Using this spherical reference, template matching was performed on binned (IMOD bin4, 13.68 Å/pix) tomograms using PyTOM. The top 600 hits (as determined by a custom Matlab script) were extracted from each of the unbinned tomograms and subjected to an initial classification in Relion 2.1 (T = 4; 25 iterations with CTF correction) allowing for up to 16 classes^52,53^. The two classes identified as SSU processome and LSU precursor were separated and refined individually, yielding the SSU and LSU precursor global averages depicted in Supplementary Fig. 2.

### Sphere fitting

From a selection of suitable tomograms where enough of the “nucleolar ring” was present (i.e. top or complete cuts; compare Supplementary Fig. 2), spheres were fit to the positions of the nucleolar SSU and LSU precursors using a custom Matlab (MathWorks) script. In brief, from an initial guess for center and radius (mean of all positions and mean distance to this center), a spherical equation was fit using a least square fit. Outlier particles (±1 standard deviation by distance to the calculated center), were then removed and the fit was repeated. This procedure was repeated until no more changes were observed and the fit which the highest number of points retained was taken.

### Density measurements

2x binned (IMOD bin 4, 13.68 Å/pixel) were denoised using neuronal networks (TOPAZ^50^) and filtered using a Gaussian kernel (Matlab imgaussfilt3, sigma = 3 pixel).

Denoised tomograms were normalized to a mean of zero and a standard deviation of one. The threshold for binarization was determined for every tomogram by measuring the mass of the densities at coordinates determined by template matching for the pre-ribosomal particles. The threshold was decrease until the mean mass of all detected subtomograms was ~4.5 MDa, and then applied to the entire tomogram for binarization. Binarized volumes were segmented using Matlab’s regionprops3, yielding xyz coordinates and the number of voxels for each object. For illustration, one such volume is shown in Fig. 2A (bottom), where densities were colored according to size in Chimera^54^. The corresponding mass of each subvolume was calculated based on voxel size and the protein density (1.3 g cm^−3^). Using aforementioned sphere fits, a distance to the center of the nucleolus was calculated for each x,y,z position. To make measurements comparable, all distances were then normalized by the radius of the determined spheres. Based on the mass and the normalized distance two types histograms were calculated:

#### One-dimensional histogram

Objects were grouped according to the distance and the mean mass was calculated for each bin (Fig. 2A).

#### Two-dimensional histogram

Objects were grouped according to mass and normalized distance. For each bin the protein density was computed (Fig. 2B).

### Probability density functions

PDFs were calculated using Matlab’s fitdist (normal kernel) function and pdf command.

### Template matching using the truncation strategy and sub-classification

To ensure purely data-driven template matching, two truncated references were generated from the initial SSU processome structure (filtered to 40 Å resolution; 13.68 Å/pix) by computationally removing density using a spherical mask as indicated in Supplementary Fig. 4^55^. Two separate rounds of template matching were then performed in PyTOM^51^ using these references. In order to over-pick, the 400 highest scoring hits were extracted from each tomogram and subjected to an initial classification using 40 classes at bin 4 (13.68 Å/pix) in Relion 2.1 (T = 4; 25 iterations with CTF correction) to check for re-growth of the removed densities. Hits were retained if a particle had a corresponding point in both lists within ±4-pixel distance of one another. This cleaned motive list was then used to extract particle sub-volumes from the twofold binned or unbinned tomograms (6.96 and 3.48 Å/pix pixel, respectively). Subtomograms were first averaged and then subjected to 3D sub-classification with six classes in Relion 2.1 (T = 4; 25 iterations with CTF correction), allowing for empty classes and yielding initial SSU processome class averages 1–3 after similarity-based tree clustering (Supplementary Fig. 5C)^56^. Final relative class abundance and classification reproducibility was determined separately (see below). Differentiation into the individual experimental states (log, stationary, DAZ-treated, synchronized-dark; see above) was performed after resolving the tomogram names. Class occupancies are reported as percental fraction within the respective state. The LSU precursor was processed analogously.

### Classification reproducibility

Subtomograms subjected to classification in Relion 2.1 were also classified using multireference alignment implemented in STOPGAP^57^. First, all subtomograms were aligned in stopgap starting with shifts and angles from Relion. There were no further improvements in FSC and map quality observed after two iterations of alignment. The resulting subtomogram alignment was used as a starting point for a multireference alignment. To determine the most variable parts of the structure, initial multireference classification was performed with a global mask. Starting references for the multireference alignment were generated by randomly assigning subtomograms to ten classes with an oversampling factor of two. Simulated annealing multireference alignment was performed against these starting references for ten iterations with decreasing temperature factors from 10 to 0. The alignment was continued without simulated annealing for 30 iterations or until the class convergence (<1% of subtomograms changing class during an iteration) was obtained. A stochastic hill-climbing search algorithm was used to score class assignments. This procedure, including generating random initial references was performed three times in total. The most variable region of a structure was determined after analyzing the resulting 30 (10 ×3) structures. Another round of simulated annealing multireference classification was performed with a mask focused on this region of the average using the procedure described above. However, for this step, we performed six replicates. Class occupancies after the class convergence for each replicate were observed to be similar (Supplementary Fig. 5A). The resulting ten structures from each of these six replicates were found to form three major clusters when subjected to hierarchical clustering (Supplementary Fig. 5B). Class consensus was defined as a subtomogram assignment to the same class, five out of six times. Final averages were generated using a consensus subtomogram assignment for each of the three classes.

Additionally, we checked for cross-effects, which might influence classification results. These include orientation, defocus, and lamella thickness (Supplementary Fig. 5D–F), none of which showed any significant correlation with class assignments.

### Structural comparisons

EM maps from published structures (EMDB accession codes indicated in the figures) were re-scaled to the same pixel size as the STA averages and filtered to the same resolution (25–35 Å). After a global alignment using a fast rotational matching (FRM) algorithm, normalized cross-correlation coefficients were calculated in MatLab (MathWorks) from masked averages using custom scripts. For illustration and orientation in overviews, published molecular models were fit into the EM-densities using UCSF Chimera’s molmap and fit-in-map commands at the respective STA map resolution.

### Distance measurements

The distance of pre-ribosomes (LSU, SSU) were calculated with respect to the individual fit nucleolar centers (see ‘Sphere Fitting’). Distances are normalized with the respective mean nucleolar radius. The data was split into each individual class and treatment.

### Data visualization

Membranes were segmented using membseg2^58^ and manually improved using Amira (Thermo Fisher Scientific). Renderings and Movies of maps and models were produced in UCSF ChimeraX^59^.

### Statistical and reproducibility

P-values were calculated using a Fisher-Pitman permutation test^60^. Significance is reported as follows: *P* ≥ 0.05: NS; *P* ≤ 0.05: *; *P* ≤ 0.01: **; *P* ≤ 0.0001: ***.

### Reporting summary

Further information on research design is available in the Nature Research Reporting Summary linked to this article.

## Supplementary information

Supplementary Information

Peer Review File

Description of Additional Supplementary Files

Supplementary Movie 1

Supplementary Movie 2

Reporting Summary

## Data Availability

The data that support this study are available from the corresponding authors upon reasonable request. The subtomogram averages generated in this study were deposited in the EMDB database under accession numbers EMD-11046, 11047 and 11048 for the LSU Classes 1–3 and EMD-11043, 11044 and 11045 for the SSU Classes 1–3, respectively. Source data are provided with this paper.

## Source data

Source Data

## Code availability

Any code used in this study is available from the authors upon request. An improved version of the pre-processing pipeline is now available on github as TOMOMAN [https://github.com/williamnwan/TOMOMAN.git].
