# Supplementary material for: In situ cryo-electron tomography reveals gradient organization of ribosome biogenesis in intact nucleoli
Source: Nat Commun. 2021 Sep 10;12:5364. doi: 10.1038/s41467-021-25413-w (PMC8433212; doi:10.1038/s41467-021-25413-w)
Supplement: Supplementary file 1 — Supplementary Information [file 41467_2021_25413_MOESM1_ESM.pdf]

Supplementary Materials for

In situ Cryo-Electron Tomography Reveals Gradient Organization of Ribosome Biogenesis in Intact Nucleoli.

Authors: Philipp S. Erdmann[*1,2], Zhen Hou[1#], Sven Klumpe[1#], Sagar Khavnekar[1#], Florian Beck[1], Florian Wilfling[1,3], Jürgen M. Plitzko[1] and Wolfgang Baumeister[*1]

Affiliations:

[1] Max Planck Institute of Biochemistry, Martinsried, Germany.

[2] Fondazione Human Technopole, Milano, Italy.

[3] Max Planck Institute of Biophysics, Frankfurt, Germany.

[*] Corresponding Authors

[#] these authors contributed equally

Please address correspondence to:

erdmann@biochem.mpg.de

baumeist@biochem.mpg.de

This PDF file includes:

Supplementary Figures 1 to 7.

Supplementary Figures:

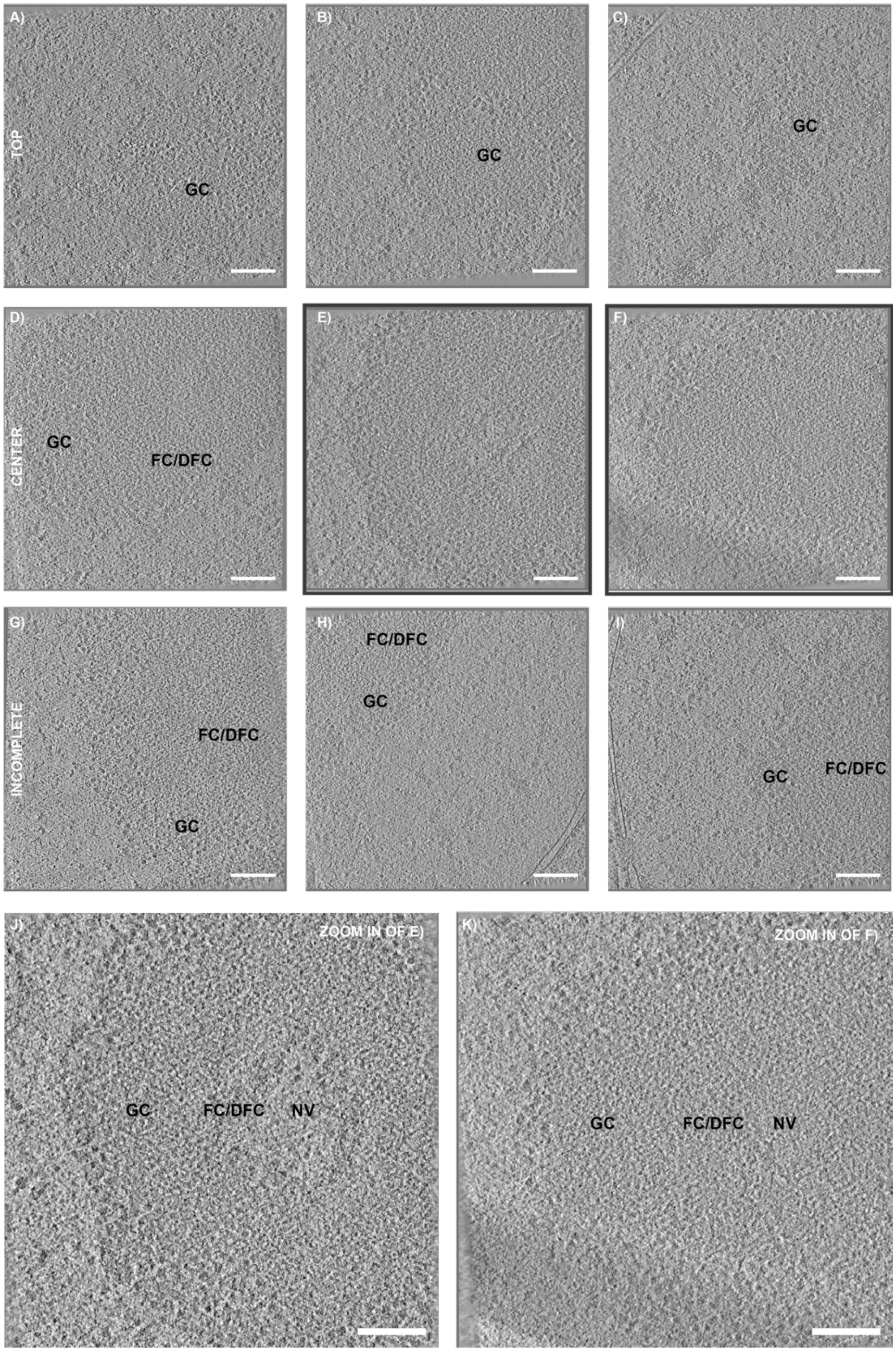

Supplementary Figure 1. Representative nuclear *C. reinhardtii* cryo-tomogram slices. Tomograms (n = 85 total) contain *Chlamydomonas* nucleoli either as top (n = 12; A-C), central (n = 24; E-F), or incomplete cuts (n = 49; G-I). Zoom-in views (J-K) of central cuts E and F show the expected tripartite organization of fibrillar component (FC), dense

fibrillar component (DFC) and granular component (GC). Additionally, nucleolar vacuoles (NV) are occasionally seen. Scale bars 200 nm.

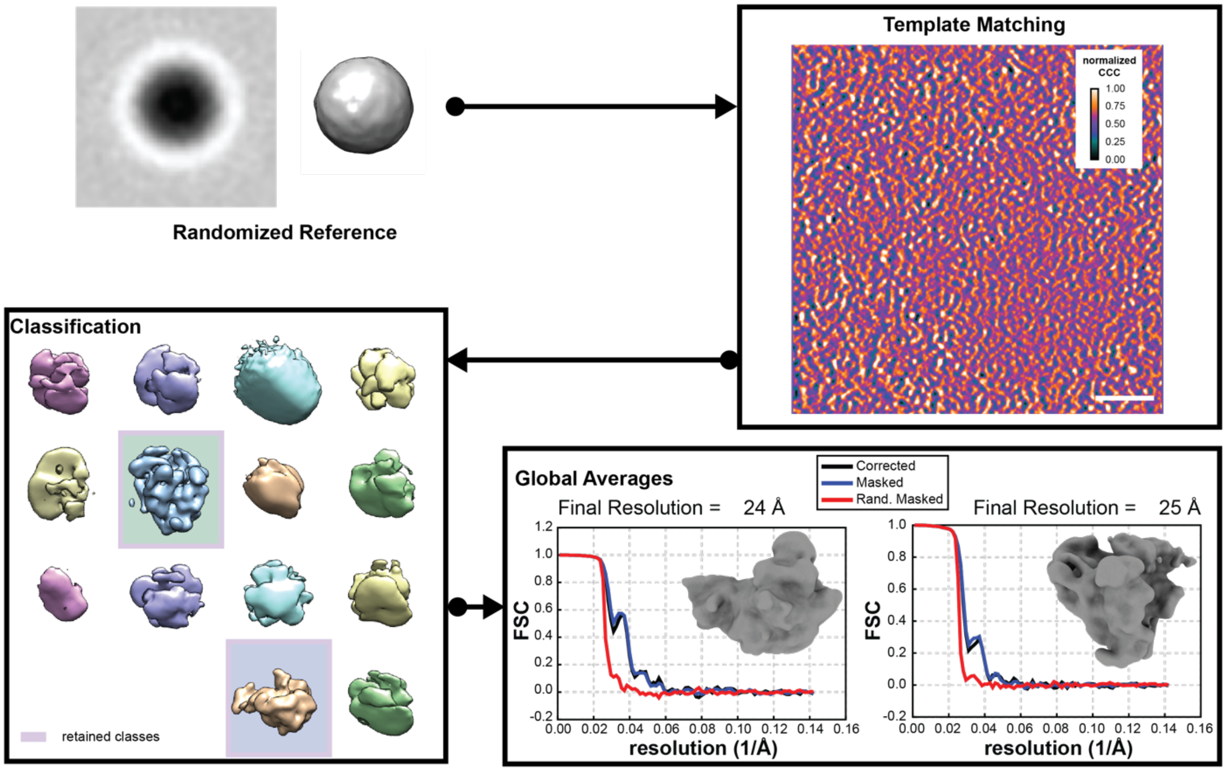

Supplementary Figure 2. Mitigating Template Bias Pt. 1: Unstructured references yield SSU and LSU precursors as main nucleolar particles. A featureless reference is constructed from 300 manually picked particles, which are back projected with random angular information, yielding a spherical template. After template matching, the initial hits are subjected to a 3D classification, again using a featureless sphere as starting reference. The two major particle classes obtained can be assigned to precursors of both the small and large ribosomal subunit, and averaged independently, yielding medium resolution averages at 24 Å and 25 Å, respectively (FSC = 0.143 criterion). CCC: cross correlation coefficient; SSU: Small Subunit precursor; LSU: Large Subunit precursor; FSC: Fourier Shell Correlation. Scale bar 200 nm.

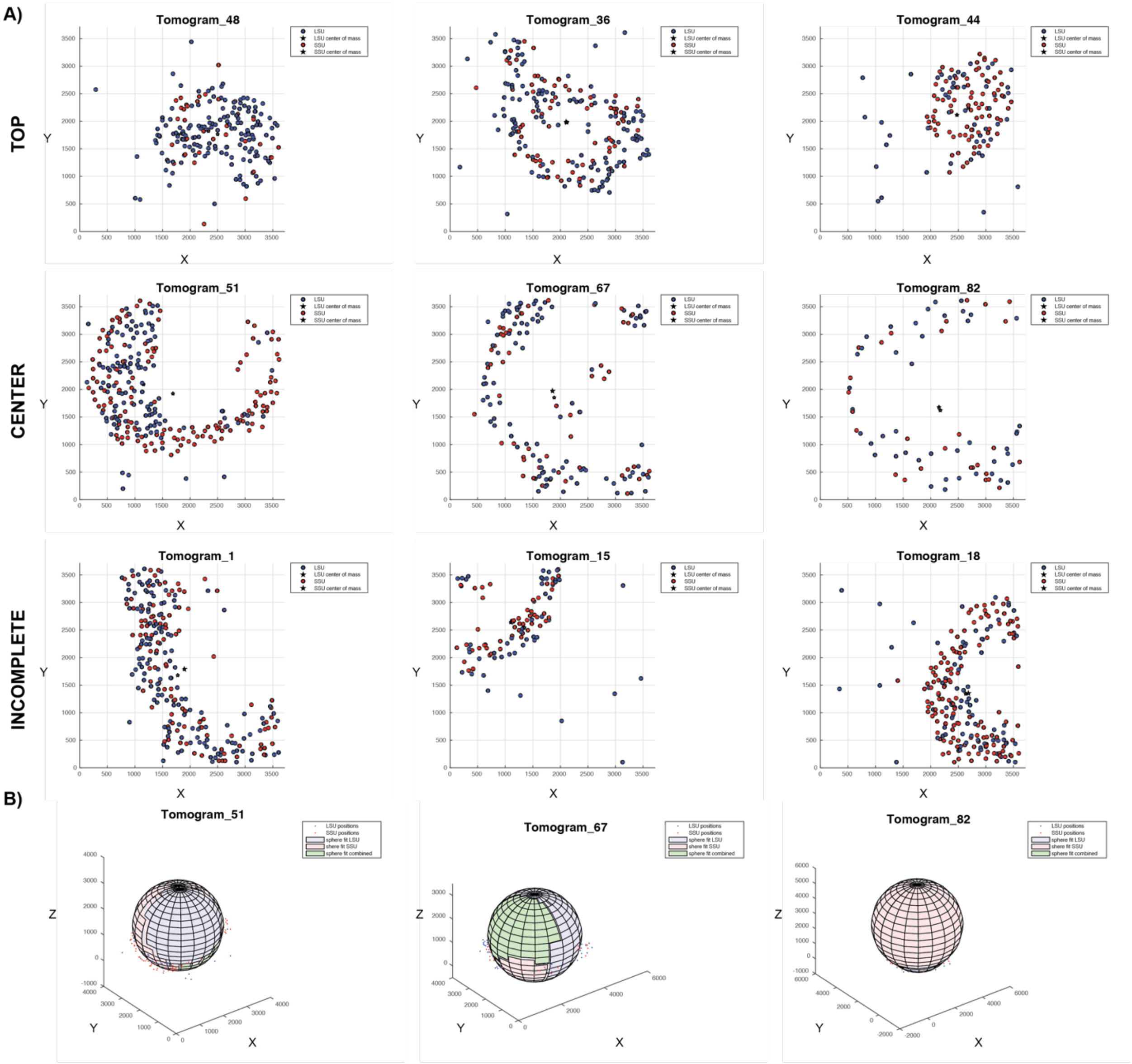

Supplementary Figure 3. Spheres can be fit to combined positions of SSU and LSU precursors. A) Positions of ribosomal precursors can be mapped back into their respective tomograms (representative tomograms from Supplementary Fig. 1). LSU precursor in blue, SSU precursor in red are shown as z-projections. B) A sphere equation can be fit to the pre-ribosome positions, especially for the center- (middle row), but also for some of the incomplete-cut tomograms (bottom row), yielding both an estimate for the size, as well as the center of the nucleoli. SSU: Small Subunit; LSU: Large Subunit.

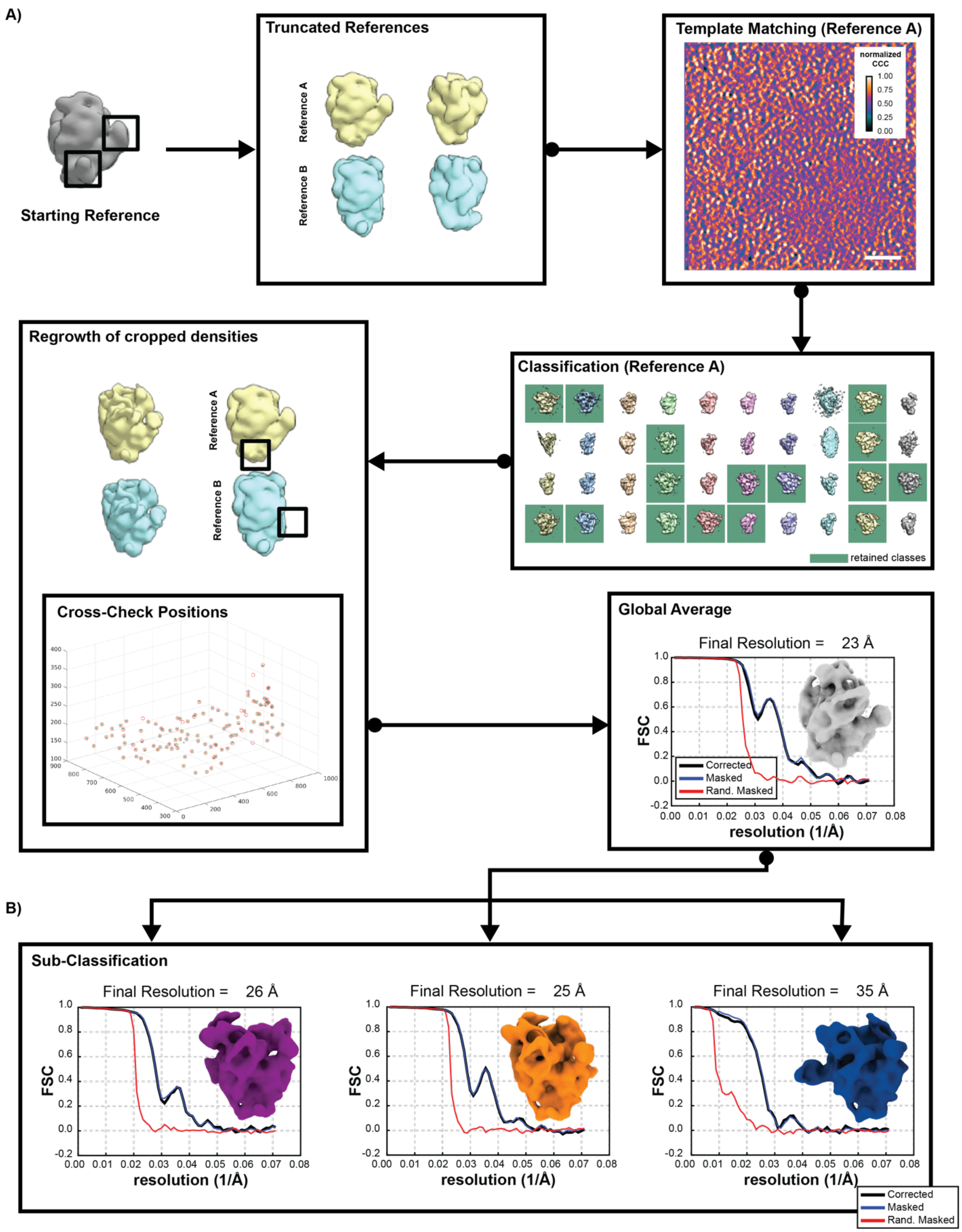

Supplementary Figure 4. Mitigating Template Bias Pt. 2: Refined template matching and sub-classification strategy reveals three distinct SSU processome classes. A) The low-pass filtered SSU reference is computationally truncated in two separate positions and used in template matching. Following 3D classification, only classes that regrow

these obligate densities are retained. For enhanced selectivity, positions from both references can be compared and only hits present in both lists are kept. B) Following 3D sub-classification, three distinct SSU processome classes ranging from 25 Å to 36 Å resolution (FSC = 0.143 criterion) are obtained. CCC: cross correlation coefficient; SSU: Small Subunit precursor; LSU: Large Subunit precursor; FSC: Fourier Shell Correlation.

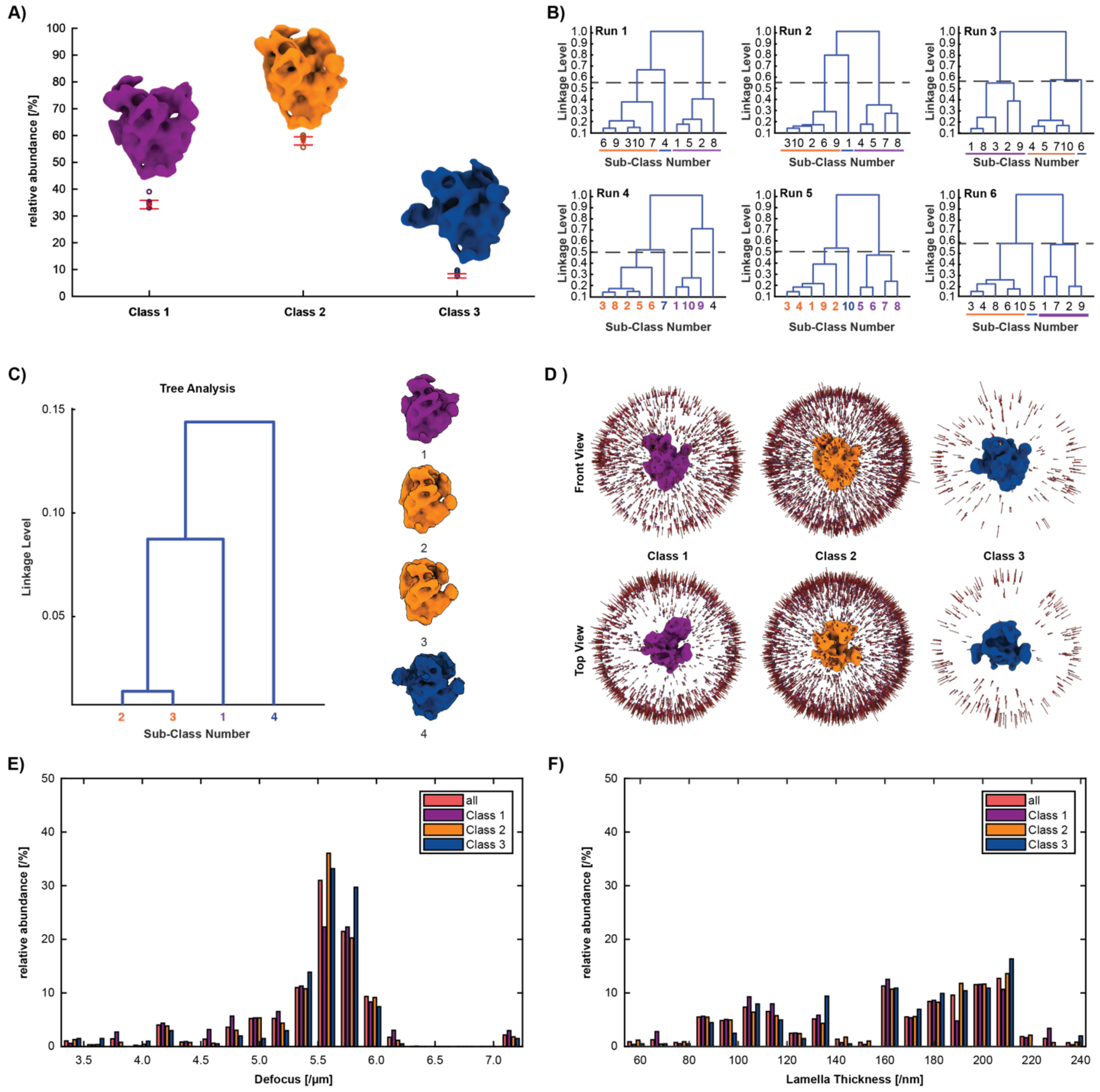

Supplementary Figure 5. SSU Processome Classification Robustness and Cross-Effect Analysis. A) Bar plot showing occupancies for three classes over six independent classification runs using a cross correlation-based approach. Class consensus for individual sub-volumes was defined as an assignment to the same class in at least five out of six runs. Mean class occupancy is shown as red cross ± STD, individual points as circles. Final averages were obtained from consensus sub-volumes. B) Hierarchical clustering dendrograms for each of the six runs. Dashed lines

show clustering threshold determined after visual inspection of volumes. In all six cases, three major structural states were identified among 10 classes. Clustered classes are depicted using the color scheme used in A. (Noise class 4. in run 4 was discarded). C) Representative tree-cluster of a Relion 2.1 3D classification with up to six classes, two of which came out empty and were discarded. Structures and relative abundance obtained by this maximum-likelihood-based classification are comparable with the max-CC approach of A-B. D) Angular distribution plots (Relion) show no preferred orientation for any of the three SSU processome classes, albeit a lower angular sampling for Class 3, the least abundant class. E-F) Likewise, there is no statistically significant correlation between class assignments and defocus (E), or the lamella thickness (F). Source data are provided as a Source Data file.

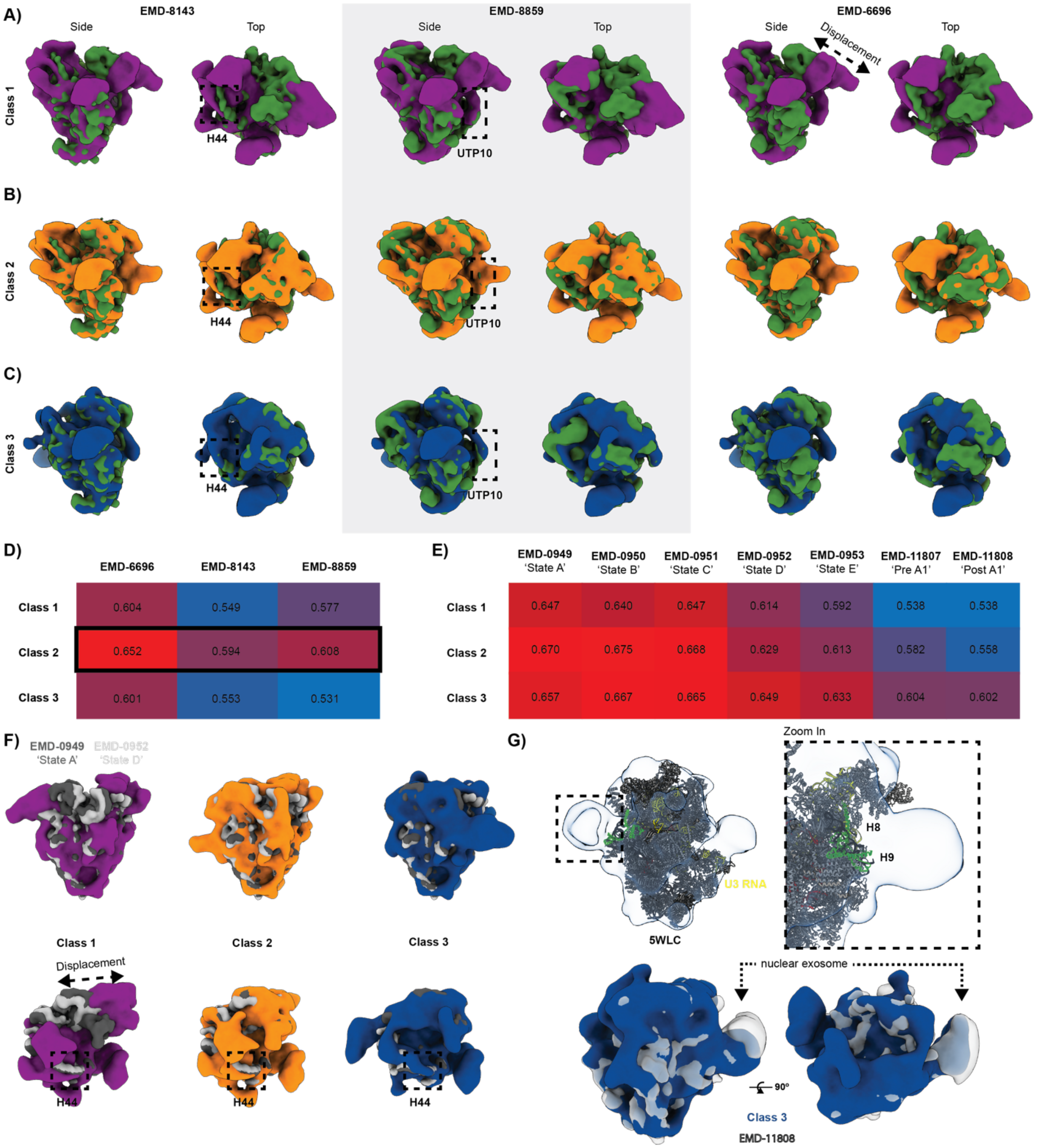

Supplementary Figure 6. Comparing the *in situ* EM maps to published densities suggests different maturation states - regardless of source organism. A-C) Global alignment of published SPA averages (EMD-8143[1], EMD-8859[2], and EMD-6696[3]) against the in situ processome Classes 1-3 provides an overall good fit, especially with Class 2. The out of place 5' domain of Class 1 is clearly visible for all three published EM maps.[1–3] D-E) Similarity between published and the *in situ* STA averages as reported by normalized cross-correlation coefficients calculated from aligned STA maps and pixel size- as well as resolution-adjusted published densities suggests a maturation from Class 1 to 2, and 3.[4,5] F) This is quite apparent when comparing the *in situ* maps with an early ("State A") and a late ("State D") stage SSU processome.[4] G) Alignment and comparison with the published map EMD-11808[5] confirms binding of the nuclear

exosome and thereby identifies SSU processome Class 3 as a late stage intermediate. Helix 8 (H8), Helix 9 (H9), Helix44 (H44) and UTP10 are marked for orientation. SSU: Small subunit precursor.

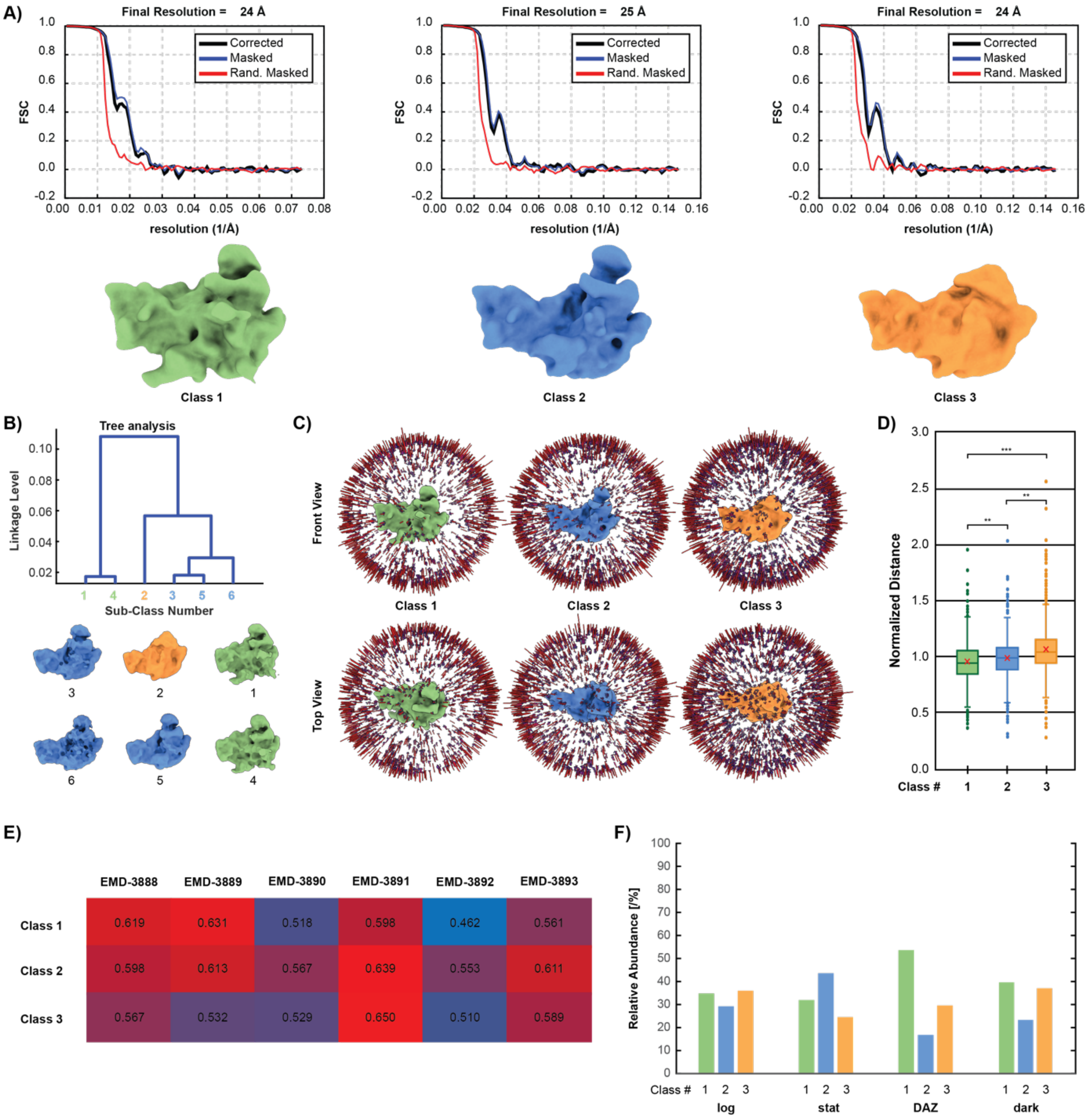

Supplementary Figure 7. A refined template matching and sub-classification strategy reveals three distinct LSU precursors and their dynamics. A) Using a similar strategy as for the SSU processome, three distinct LSU precursors are obtained after 3D sub-classification.[6] B) Representative tree cluster of a classification run. C) Angular distribution for all three large subunit precursors. D) Just like the SSU processome, LSU Class1-3 also show a maturation-correlated localization pattern (n = 1820 subtomograms; two-sided Fisher-Pitman permutation test of mean: $p_{1,2}$ = 1E-06, $p_{1,3}$ = 8.84E-04, and $p_{2,3}$ = 1.59E-04; data are represented as boxplots where *the red cross indicates the mean,* the middle line is the median, the lower and upper hinges correspond to the first and third quartiles*,* top and bottom whiskers indicate maximum and minimum, respectively. Circles show outlier points.). E) Similarity between

published[6] and the *in situ* STA maps as reported by normalized cross-correlation coefficients of the aligned maps (at same pixel size and resolution). F) The relative abundance of the large subunit precursors varies upon different stimuli. Especially diazaborine (DAZ) treatment shows a significant increase of LSU precursor Class 1 relative to normal growth (log), stationary (stat), or a dark-adapted synchronized cell culture (dark). FSC: Fourier Shell Correlation. Source data are provided as a Source Data file.

Supplementary Material Literature.

1. Kornprobst, M., Turk, M., Kellner, N., et al., Architecture of the 90S Pre-ribosome: A Structural View on the Birth of the Eukaryotic Ribosome. *Cell*, 2016, **166**, 380–393.
2. Barandun, J., Chaker-margot, M., Hunziker, M., Molloy, K. R., Chait, B. T., and Klinge, S., The complete structure of the small-subunit processome. *Nat. Struct. Mol. Biol.*, 2017, **24**.
3. Sun, Q., Zhu, X., Qi, J., et al., Molecular architecture of the 90S small subunit pre-ribosome. *Elife*, 2017, **1**, 1–28.
4. Du, Y., An, W., Zhu, X., Sun, Q., Qi, J., and Ye, K., Cryo-EM structure of 90S small ribosomal subunit precursors in transition states. *Science (80-.).*, 2020, **369**, 1477–1481.
5. Lau, B., Cheng, J., Flemming, D., et al., Structure of the Maturing 90S Pre-ribosome in Association with the RNA Exosome. *Mol. Cell*, 2021, 1–11.
6. Kater, L., Thoms, M., Barrio-Garcia, C., et al., Visualizing the Assembly Pathway of Nucleolar Pre-60S Ribosomes. *Cell*, 2017, **171**, 1599-1610.e13.
