# Supplementary material for: In situ cryo-electron tomography reveals gradient organization of ribosome biogenesis in intact nucleoli
Source: Nat Commun. 2021 Sep 10;12:5364. doi: 10.1038/s41467-021-25413-w (PMC8433212; doi:10.1038/s41467-021-25413-w)
Supplement: Supplementary file 3 — Description of Additional Supplementary Files [file 41467_2021_25413_MOESM3_ESM.pdf]

Description of Additional Supplementary Files

File Name: Supplementary Movie 1
Description: **Visualization of a representative C. reinhardtii tomogram**. After reconstruction, segmentation, and template matching, pre- ribosomal particles can be visualized on the raw data. Pre-60S and SSU processome are clearly visible and show mostly an orbital localization around the nucleolus. Based on their positions, the total nucleolar volume can be estimated and visualized.

File Name: Supplementary Movie 2
Description: **Animation of SSU Processome Classification.** Based on the global average, three major small subunit precursor classes are obtained after 3D classification. A morph between the structures suggests a steady compactification from Class 1 to 2, and 3.
