# Supplementary material for: In situ cryo-electron tomography reveals gradient organization of ribosome biogenesis in intact nucleoli
Source: Nat Commun. 2021 Sep 10;12:5364. doi: 10.1038/s41467-021-25413-w (PMC8433212; doi:10.1038/s41467-021-25413-w)
Supplement: Supplementary file 6 — Reporting Summary [file 41467_2021_25413_MOESM6_ESM.pdf]

nature research

Corresponding author(s): Philipp S Erdmann, Wolfgang Baumeister

Last updated by author(s): Mar 15, 2021

Reporting Summary

Nature Research wishes to improve the reproducibility of the work that we publish. This form provides structure for consistency and transparency in reporting. For further information on Nature Research policies, see our Editorial Policies and the Editorial Policy Checklist.

Statistics

For all statistical analyses, confirm that the following items are present in the figure legend, table legend, main text, or Methods section.

n/a	Confirmed	
☐	☒	The exact sample size (n) for each experimental group/condition, given as a discrete number and unit of measurement
☐	☒	A statement on whether measurements were taken from distinct samples or whether the same sample was measured repeatedly
☐	☒	The statistical test(s) used AND whether they are one- or two-sided<br>*Only common tests should be described solely by name; describe more complex techniques in the Methods section.*
☒	☐	A description of all covariates tested
☒	☐	A description of any assumptions or corrections, such as tests of normality and adjustment for multiple comparisons
☐	☒	A full description of the statistical parameters including central tendency (e.g. means) or other basic estimates (e.g. regression coefficient) AND variation (e.g. standard deviation) or associated estimates of uncertainty (e.g. confidence intervals)
☐	☒	For null hypothesis testing, the test statistic (e.g. F, t, r) with confidence intervals, effect sizes, degrees of freedom and P value noted<br>*Give P values as exact values whenever suitable.*
☒	☐	For Bayesian analysis, information on the choice of priors and Markov chain Monte Carlo settings
☒	☐	For hierarchical and complex designs, identification of the appropriate level for tests and full reporting of outcomes
☒	☐	Estimates of effect sizes (e.g. Cohen's d, Pearson's r), indicating how they were calculated

Our web collection on statistics for biologists contains articles on many of the points above.

Software and code

Policy information about availability of computer code

Data collection	open source: SerialEM 3.7, Motioncor2 1.2.2
Data analysis	open source: IMOD 4.10.10, CTFFIND 4.1.8, membseg2, PyTOM 0.97, Relion 2.1/3.0, STOPGAP 0.7.1, TOPAZ 0.2.4, ChimeraX 0.92; closed source: Matlab R2018a, Amira 2019.2

For manuscripts utilizing custom algorithms or software that are central to the research but not yet described in published literature, software must be made available to editors and reviewers. We strongly encourage code deposition in a community repository (e.g. GitHub). See the Nature Research guidelines for submitting code & software for further information.

Data

Policy information about availability of data

All manuscripts must include a data availability statement. This statement should provide the following information, where applicable:

- Accession codes, unique identifiers, or web links for publicly available datasets
- A list of figures that have associated raw data
- A description of any restrictions on data availability

Subtomogram averages have been deposited on EMDB under accession numbers EMD-11046, 11047, and 11048 for the LSU Classes 1-3 and EMD-11043, 11044, and 11045 for the SSU Classes 1-3, respectively. They are linked in the text with their unique accession numbers and URLs.

Field-specific reporting

Please select the one below that is the best fit for your research. If you are not sure, read the appropriate sections before making your selection.

[x] Life sciences [ ] Behavioural & social sciences [ ] Ecological, evolutionary & environmental sciences

For a reference copy of the document with all sections, see nature.com/documents/nr-reporting-summary-flat.pdf

Life sciences study design

All studies must disclose on these points even when the disclosure is negative.

Sample size	85 tomograms were used in this study. The sample size (85 tomograms from 85 cells) was chosen because of the practical limitations (throughput) of the methodology used. It was sufficient to interpret the data in a robust manner (classification) also according to previous publications (see for example Table S2 in Watanabe et al. Cell 2020 or Supplementary Table 1 in Kiesel et al. Nat Struct Mol Biol 2020).
Data exclusions	No data was excluded from the analysis.
Replication	The data is compiled from 10 individual batches of cells that were plunge-frozen and processed sequentially (split into log, stat, dark, blocked; see main text). All attempts at replication were successful.<br>Electron microscopy data are from 85 individual cells. Each cell can therefore be considered as a biological replicate.
Randomization	Cells were selected at random for cryo-EM experiments without bias toward location, size, or other parameters.
Blinding	Tomogram names were hashed/renamed before processing to hide their state/origin and resolved into the individual states (log; stationary; synchronized; DAZ-treated) only after final classification was done.

Reporting for specific materials, systems and methods

We require information from authors about some types of materials, experimental systems and methods used in many studies. Here, indicate whether each material, system or method listed is relevant to your study. If you are not sure if a list item applies to your research, read the appropriate section before selecting a response.

Materials & experimental systems

n/a	Involved in the study
[x]	[ ] Antibodies
[ ]	[x] Eukaryotic cell lines
[x]	[ ] Palaeontology and archaeology
[x]	[ ] Animals and other organisms
[x]	[ ] Human research participants
[x]	[ ] Clinical data
[x]	[ ] Dual use research of concern

Methods

n/a	Involved in the study
[x]	[ ] ChIP-seq
[x]	[ ] Flow cytometry
[x]	[ ] MRI-based neuroimaging

Eukaryotic cell lines

Policy information about cell lines

Cell line source(s)	CC-3994 mat3-4 mt+; ChlamyCollection.org: https://www.chlamycollection.org/product/cc-3994-mat3-4-mt/
Authentication	no further authentication done
Mycoplasma contamination	not applicable (plant cell line)
Commonly misidentified lines (See ICLAC register)	not applicable
